# Patient-Specific Modeling of Diffuse Large B-Cell Lymphoma

**DOI:** 10.3390/biomedicines9111655

**Published:** 2021-11-10

**Authors:** Kirsten Thobe, Fabian Konrath, Björn Chapuy, Jana Wolf

**Affiliations:** 1 Mathematical Modelling of Cellular Processes, Max Delbrück Center for Molecular Medicine, 13125 Berlin-Buch, Germany; kirsten.thobe@mdc-berlin.de (K.T.); fabian.konrath@mdc-berlin.de (F.K.); 2 Department of Hematology and Medical Oncology, University of Göttingen, 37075 Göttingen, Germany; bjoern.chapuy@med.uni-goettingen.de; 3 Department of Hematology, Oncology and Cancer Immunology, Berlin Medical Center Charité, 12203 Berlin, Germany; 4 Department of Mathematics and Computer Science, Free University Berlin, Arnimallee 14, 14195 Berlin, Germany

**Keywords:** cancer, patient-specific treatment, personalized medicine, logical modeling, DLBCL, signaling networks

## Abstract

Personalized medicine aims to tailor treatment to patients based on their individual genetic or molecular background. Especially in diseases with a large molecular heterogeneity, such as diffuse large B-cell lymphoma (DLBCL), personalized medicine has the potential to improve outcome and/or to reduce resistance towards treatment. However, integration of patient-specific information into a computational model is challenging and has not been achieved for DLBCL. Here, we developed a computational model describing signaling pathways and expression of critical germinal center markers. The model integrates the regulatory mechanism of the signaling and gene expression network and covers more than 50 components, many carrying genetic lesions common in DLBCL. Using clinical and genomic data of 164 primary DLBCL patients, we implemented mutations, structural variants and copy number alterations as perturbations in the model using the CoLoMoTo notebook. Leveraging patient-specific genotypes and simulation of the expression of marker genes in specific germinal center conditions allows us to predict the consequence of the modeled pathways for each patient. Finally, besides modeling how genetic perturbations alter physiological signaling, we also predicted for each patient model the effect of rational inhibitors, such as Ibrutinib, that are currently discussed as possible DLBCL treatments, showing patient-dependent variations in effectiveness and synergies.

## 1. Introduction

Diffuse large B-cell lymphoma (DLBCL) is the most common lymphoid malignancy in adults. Despite exhibiting a large molecular heterogeneity with recognized subtypes, there is currently a common immunochemotherapy-based treatment for all patients [1]. While this treatment cures approx. 60–70% of patients, it leaves non-responders with relapsed or refractory disease and limited treatment options.

DLBCL arises from antigen-exposed B-cells that are undergoing somatic hypermutations in the germinal center (GC) in order to generate highly specific antibodies towards these antigens [2]. The genomic instability occurring in the GC requires to activate BCL6 to transiently tolerate the genotoxic stress and to physiologically initiate the dark zone (DZ) of the germinal center [3,4]. This process is followed by a rigorous selection based on competition for survival signals and lower proliferation rates in the light zone (LZ), such that only B-cells with optimal antigen-antibody binding survive while the remainders undergo apoptosis due to lack of signals [5]. The survival signals are triggered by antigen B-cell receptor (BCR) interaction, as well as T follicular helper cell interaction with CD40 receptor and IL21/IL4 receptors [6]. The pathways transmitting signals from the receptors to crucial genes for differentiation and survival encompass PI3K, MAPK, and NF-κB signaling as well as JAK/STAT signaling. If a cell receives and successfully processes all of these signals, it differentiates to a plasma cell, ensuring survival outside of the germinal center. Notably, during malignant transformation, antigen-exposed B-cells acquire genetic lesions within the above-described pathways (and others, see [7,8]) that ontogenically activate pro-survival pathways for these B-cells.

**Figure 1:**
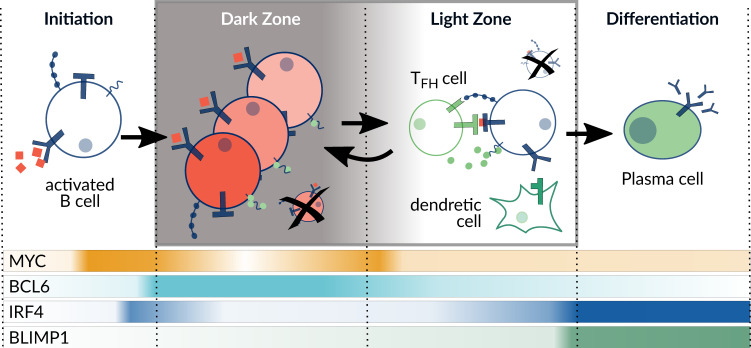
Overview of the germinal center reaction. The differentiation process comprises four different stages with specific expression levels of selected marker genes (MYC, BCL6, IRF4, BLIMP1) indicated on the bottom. Adapted from Basso et al. (2015) [1].

Research in the last decade highlighted differentiation markers that are active during four defined stages of the GC reaction: initiation, dark zone, light zone, and plasma cell differentiation (Figure 1) [1]. To initiate the GC reaction, a burst in MYC expression is essential just before entering the dark zone, where it enables the high proliferation rate of B-cells. However, MYC expression declines constantly, which leads to the suggestion that the MYC level acts as a timer of the DZ [9]. To date, it is only partly understood which combination of receptor activation is necessary for the decision to transfer to a specific GC stage [10]. For example, NF-κB controls the differentiation genes *BCL6* and *BLIMP1,* and cells with compromised NF-κB signaling were shown to have severely impaired GCs [11]. NF-κB can be activated by BCR and CD40 signaling, while the specific timing and interplay of these receptors and their ligands at the different stages is incompletely understood. The understanding of the network connecting receptors, signaling, and marker gene expression is of high relevance, since many of the involved pathways are reported to be deregulated in DLBCL.

Based on the cell-of-origin, DLBCL have been classified into two subtypes, where GCB-like DLBCL are similar to light zone B-cells and are described to be driven by genetic alterations of *CREBBP* and *EZH2* [12]. ABC-like DLBCL present a highly active NF-κB pathway like differentiating cells, which cannot reach the plasma or memory cell fate. Here, the most prominent altered genes are *CARD11*, *MYD88, TNFAIP3*, and *CD79B* [13]. Especially DLBCLs of type ABC show less responsiveness upon standard treatment and are therefore the focus of further studies [14]. However, there are also patients with a GCB-like profile with an unfavorable outcome, suggesting that transcriptional signatures only partially capture the features relevant for outcome prediction [13]. More recently, comprehensive sequencing studies of DLBCL facilitated a more refined subclassification of the heterogeneous genomic profiles of DLBCL patients [7,8]. Here, statistical data analysis approaches such as clustering are applied with the aim of identifying treatment groups and thereby improving the patient outcome. A different perspective to statistics-based stratification gives patient-specific modeling, where genomic profiles are combined with pathway models. These pathway models are developed from biological insights into the functioning of the underlying molecular processes. Patient-specific modeling enables a more detailed view on differences between patients on the molecular level in order to improve the understanding of heterogeneity of the disease and to potentially aid optimal treatment prediction [15,16,17,18,19].

Here, we develop a computational model that simulates the survival regulation of B-cells in the GC and allows to create patient-specific models based on genome data to study the heterogeneity of DLBCL on a mechanistic pathway level. Since the available data is mostly of qualitative nature, we chose a qualitative modeling approach, logical modeling. This approach has already been proven to be a powerful tool in the description and analysis of cell fate decisions involved in development and cancer [20,21,22,23]. Based on the genome data, we aim for constructing patient-specific models as perturbations of a wild type B-cell model. With these models, we want to capture individual combinations of mutations, copy-number alterations and structural variants of more than 50 components. Moreover, we want to explore, in detail, the role of perturbations in the NF-κB pathway on the oncogenic model behavior. Finally, by simulating the response of each model to different drugs and drug combinations, we are able to predict patients and groups of patients to be sensitive or resistant to a drug.

## 2. Materials and Methods

### 2.1. Logical Modeling

Logical models for biological system were first formalized by Kauffman (1969) [24], where the topology of the system is defined as a directed graph R=(V, E,t), called interaction graph, with V={1, …, n} as nodes that represent the components of the system, e ∈E⊆V×V as edges representing interactions between components and t:E→ℕ assigning thresholds to the edges. Here, the components represent receptors, genes, and protein complexes, whereas interactions indicate binding, transport, post-transcriptional modifications, or expression. The components can adopt so-called activity levels, for Boolean models either 0 or 1 is assigned to each component B={0, 1}, indicating an inactive or active state, respectively. The state s of a system is defined if every component is set to an activity level, thus s:V→{0, 1},∀v∈V:s(v)∈B, where the sequence presents the same ordering as V. This formalism was extended to logical models, where more discrete activity levels can be defined. In this model, some components are ternary, and thus can reach an activity level of 2. The edges of the model are additionally assigned with a threshold t, which determines at which level an edge becomes effective.

A component is regulated by its predecessor nodes, which determine when a component becomes active. If there is more than one predecessor, the regulation can be expressed using the logical operators ∨ (OR), ∧ (AND), and ¬ (NOT) in a formula$\;f_{V}$. The dynamic behavior of a model results from state transitions by updating the components activity over discrete time steps. Every generated trajectory finally reaches an attractor, which is a set of states that the trajectory cannot leave. In case this set of states is a single state, thus no component changes f(s)=s, a fixpoint of the system is reached. The fixpoints of a system are conserved for all updating strategies.

**Figure 2:**
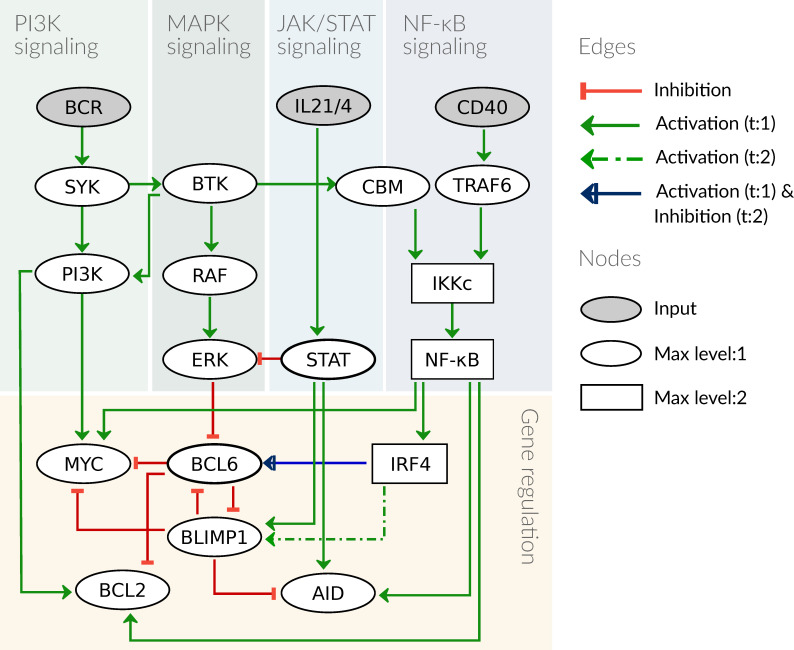
Graphical scheme of the model. The model contains four signaling pathways and a gene regulation module, with three receptors as input nodes to the system. All components are binary except for IKKc, NF-κB, and IRF4, which are ternary. The edges are mostly inhibiting or activating with a threshold of 1 (t:1), except the edge from IRF4 to BLIMP1 with a threshold of 2 and the edge from IRF4 to BCL6, which is activating if IRF4 = 1 and inhibiting if IRF4 = 2.

Here we build a logical model for the processes in the germinal center comprising relevant signaling pathways and gene expression processes (shown in Figure 2); biological details will be given in Section 3.1. The model building process was done in GINsim [25]. The model is analyzed with respect to its fixpoints under different stimulations, Section 3.2, and then specified for patient-specific data, see Section 2.2 and Section 3.4.

### 2.2. Patient-Specific Models Derived from Clinical Data

Our analysis is based on a recently published, comprehensive data set that genetically characterized 304 primary DLBCL patients [7]. Notably, 85% of patients in this cohort were uniformly treated with state-of-the-art therapy (rituximab-containing CHOP-like). Samples of newly diagnosed patients were used to perform whole-exome sequencing that were analyzed for copy number (CN) alterations, somatic variants, and mutations. The significance of these were evaluated (for details see [7]) and categorized for mutations as non-synonymous mutations, synonymous mutations, or no-mutation. For copy number alterations, categories are high-grade CN gain, low-grade CN gain, CN neutral as well as low-grade CN loss, and high-grade CN loss [7].

**Figure A1:**
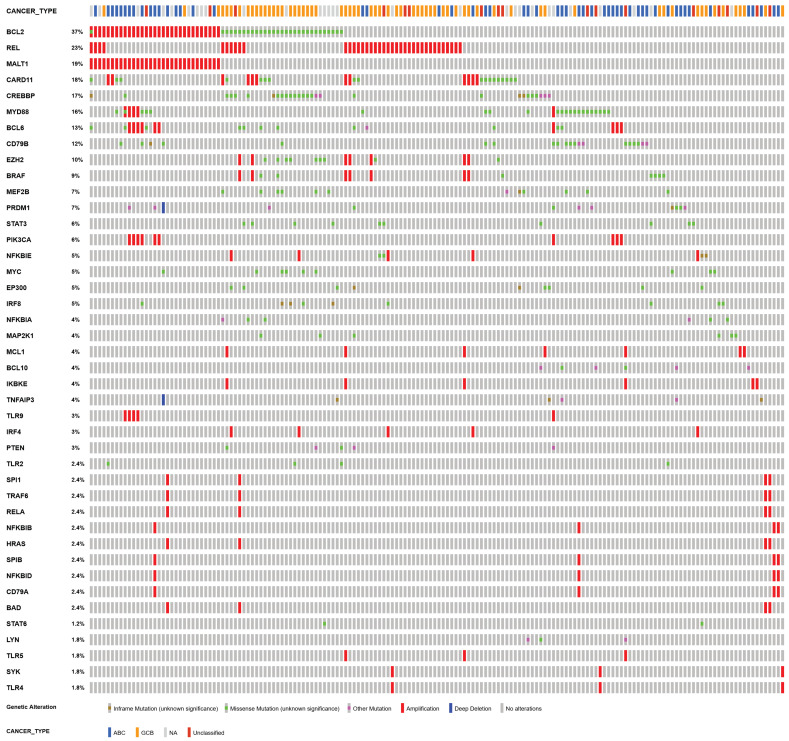
Visualization of the genetic alterations included in our analysis as Oncoprint from cBioportal [26,72]. The plot shows the 42 perturbed out of 54 covered genes as rows color coded for their alteration in the 164 patients as columns. CN deletions are shown as a blue bar, CN amplifications as a red bar. Mutations are shown as boxes, where green shows a missense mutation, brown for inframe, and pink for other mutations. On the top, the COO type for the patients are given, blue for ABC, yellow for GCB, red for unclassified, and grey if no information was available.

Based on the general model of the molecular processes of the GC reaction, we are generating patient-specific models by implementing the described genetic alteration types and levels. In our analysis, we focus on strong effects and therefore only included high-grade copy number gains and losses as well as non-synonymous mutations with an annotated non-silent effect. Moreover, we only considered perturbations of those genes that are associated with the components of our model. To this end, we used a manually curated list of genes (data not shown). These genes were then queried from the published study with the genetic alterations for each patient and presented in an Oncoprint format from cBioportal (https://www.cbioportal.org/, accessed on 4 November 2021). [26], see Figure A1.

**Figure 5:**
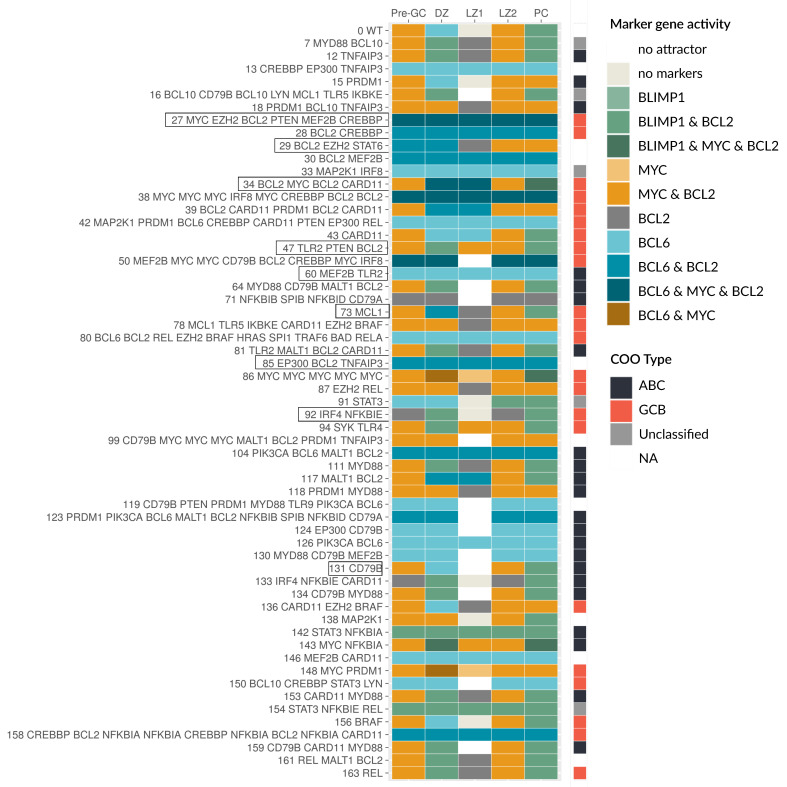
Patient-specific models show large heterogeneity in attractor patterns. A selection of patient-specific models is shown where the model ID and the perturbed genes are given on the left, ordered by model ID. The attractors are color coded for their marker gene activity, as well as the COO status.

For the implementation of the perturbations in patient-specific models, copy-number alterations are interpreted as loss of function (LOF) for deletions and as gain of function (GOF) for amplifications. Mutations and structural variants are set to GOF for oncogenes and LOF for tumor suppressors. In case the perturbation affects a component of the model directly, this effect is then implemented as a changed activity state of the component. Otherwise, the effect of the perturbation is mapped onto the subsequent model component as shown in Figure 4, taking the sign of the edge into account. Then, the implementation into the model is done by setting the affected component to either 0 or 1, for LOF and GOF respectively. Formally, this results in a new model for each patient (annotated by a model ID), due to changes in the logical function of components, which was implemented in the CoLoMoTo notebook [27]. The new models can have different attractors than the unperturbed model. In cases the perturbation affects an input node, the number of attractors will be reduced, since a specific input combination is not possible anymore as indicated by “no attractor” in Figure 5.

## 3. Results

### 3.1. Computational Model of the Germinal Center Reaction

We first develop a model for GC B-cells that might serve as physiological equivalent from which DLBCL cells arise through malignant transformation. Our basic assumption for the behavior of a GC B-cell is that it is constantly undergoing selection pressure in the form of competition for survival signals from T helper cells and antigens. For our model, this implies that without activation of receptors, the cell will not express any proliferation, survival, or differentiation markers. In contrast, activation signals from T helper cells and antigens alone or in combination provide input combinations that are specific for individual stages of the GC reaction and cause stage-specific expression of marker genes. In order to describe the critical processes involved in the regulation of marker gene expression, the model comprises PI3K, MAPK, NF-κB, and JAK/STAT signaling.

The signaling pathways are described as minimal as possible to reduce the complexity of the model. We therefore focus on pathway components that are either targeted by genetic lesions or affected by drug treatment. Thus, linear signaling chains such as RAS → RAF → MEK → ERK are reduced without affecting dynamical features of the model [28]. In Figure 2, the structure of the model is presented as a graph depicting components and interactions of the model. Logical functions of the model are given for all components that have more than one regulator as detailed in Table 1. In case there is only one regulator, the activity of a component depends on the state of its predecessor and type of interaction, i.e., activation or inhibition (for more details see Section 2.1, the full model is given in Table A1).

Active BCR signaling gives the primary survival signal for a B-cell. Upon BCR engagement by its antigen, the BCR recruits several adaptor and signaling intermediates such as kinases, LYN, and SYK, which activate PI3K and AKT. A central component of the BCR signaling is also BTK, which besides its canonical role in activating NF-κB, also modulates AKT together with SYK [29]. As a consequence, in our model we included the activation of SYK and BTK and mapped this AKT regulation by SYK and BTK on PI3K. As indicated above, BTK activates PKCβ, which subsequently promotes both MAPK activation via RAS and NF-κB signaling via a complex of CARD11, BCL10, and MALT1 (CBM) (see [44] and reference therein). We here include these regulations in the model in a reduced form: BTK activates RAF as well as the CBM complex. A central role of PI3K signaling in the GC reaction is to enable proliferation in the DZ by activating MYC.

The NF-κB pathway becomes active after the formation of the IKK complex (IKKc) comprising NEMO, IKKα, and IKKβ. Active IKKc inhibits IκB, which in turn releases the repression of NF-κB, leading to transcription of a wide array of target genes [45]. The IKK complex can be activated by different upstream receptors and kinases [46]; in GC B-cells, IKKc is activated mainly by BCR via BTK and the CBM complex as well as by CD40 via TRAF6 (Figure 2). Moreover, there are different modes of activity described for NF-κB leading to varying downstream behavior, specifically for its target IRF4. IRF4 dynamics have been shown to be essential for the decision process during differentiation, where a low and transient transcription is essential for BCL6 activity in the DZ whereas a high and permanent IRF4 activity is sufficient to drive B-cell differentiation to plasma cells [47]. In order to reflect these two different dynamics in the NF-κB pathway and its target gene IRF4, the pathway components are designed to show three different activities: no activity (0), low and transient activity (1), and high activity (2). Here, high activity results from an activation of CD40, since it was shown to be the essential stimulus for PC differentiation [36], which is mediated by high levels of NF-κB [47]. We modeled BCR stimulation to cause a low NF-κB activity, since it requires T cell help to cause PC differentiation.

T helper cells not only stimulate B-cells through the CD40 receptor, but also activate the JAK/STAT pathway through interleukin (IL) 21 and 4. These cytokines have been shown to be present in high levels in the DZ, where they activate STAT6 to block ERK from phosphorylating and thereby inhibiting BCL6. This allows for rapid and reversible high levels of BCL6 in the DZ [33,34]. STAT3 was shown to be essential for PC differentiation by forming a complex with IRF4 [48]. Finally, STATs were also shown to induce AID transcription together with NF-κB [39]. For our model, we summarize the IL21 and IL4 receptors to one component IL21/4, since they were shown to have similar functions in GC B-cells [33]. The JAK/STAT pathway is represented by the component STAT, which acts on the targets ERK, BLIMP1, and AID.

The regulation of B-cell marker genes including BCL6, BLIMP1, IRF4, and AID has been modeled by different groups [47,49,50]. We adapted our model from the logical model by Mendez and Mendoza (2016) [51] and extended the upstream regulatory pathways as described above. We added MYC and BCL2 as additional markers and reduced the number of interactions between the marker genes. In detail, BCL6 is inhibited in the GC by ERK and independently by BLIMP1, which is implemented as a logical OR connection. The activation of BCL6 was shown to follow an early burst in expression of IRF4 [52], which we implement as IRF4:1 (see Table 1). BLIMP1 is inhibited by BCL6 and requires a complex formation of STAT with high level IRF4 to become active [38]. The DZ marker AID is an enzyme that has a large number of regulators, however, we focus on regulations from components in the model. STAT6 and NF-κB have been described as transcriptional activators of AID [39], while BLIMP1 inhibits AID expression [40]. The proliferation marker MYC is critical to enable the clonal expansion in the dark zone, and its activation requires not only PI3K activation but also NF-κB signaling in GC cells [32], while an inhibition of MYC by BLIMP1 was described in differentiated B-cells [30]. Additionally, BCL6 blocks the transcription of MYC in the DZ [31], this inhibition is commonly disrupted in DLBCL. Finally, the marker BCL2 was added as a pro-survival signal, an important read-out for the selection process in the germinal center, and additionally, it is a frequent oncogene in DLBCL (see Figure A1). PI3K as well as NF-κB are described to promote survival via BCL2, where NF-κB directly acts as a transcription factor for BCL2 [43]. PI3K promotes survival through AKT by inhibiting the pro-apoptotic protein BAD, and thereby promotes activity of BCL2-family members [41,42]. This regulation is implemented as activation of BCL2 by PI3K in the model.

### 3.2. Attractors of the Model Reflect Germinal Center Stages

The model is used to simulate the different stages of B-cell differentiation in the GC as attractors of the system. Since B-cells are moving through different environments and receive specific signals to progress towards a plasma cell (Figure 1), we assume that for the duration of a stage the marker expression of a B-cell should be robust and reproducible from the input. Thus, the model was built to have GC stages as attractors, even though they should not be interpreted as long-term states since the GC reaction is a dynamical process. However, the patient-specific models are expected to be in a steady-state-like behavior, since they represent cancer cells rather than differentiating cells.

**Figure 3:**
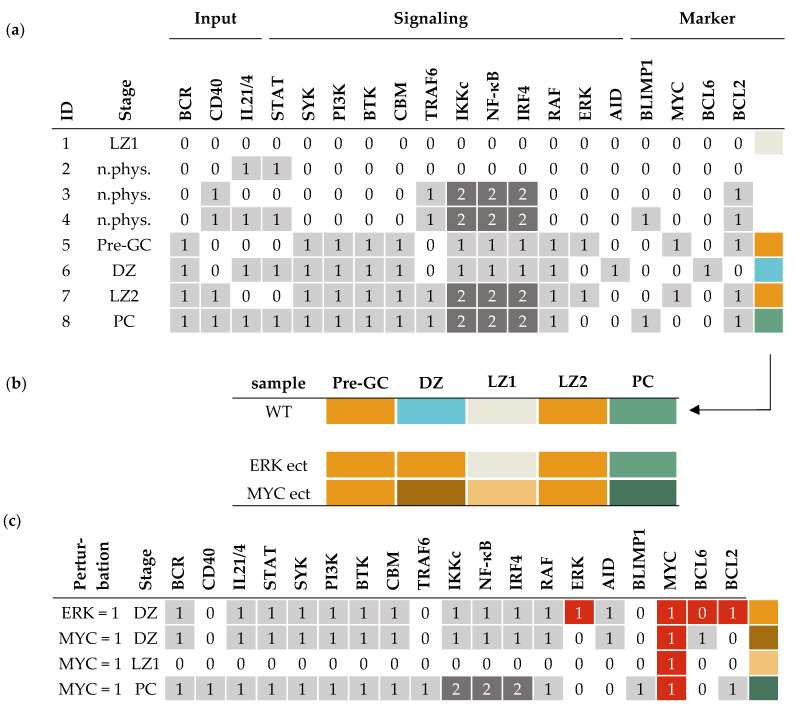
Attractors of the GC model and associated germinal center stage. (**a**) List of all attractors of the model, where each row represents an attractor reached from an input combination of BCR, CD40, and IL21/4. Activity levels of components are 0 (white), 1 (light grey) or 2 (dark grey). Stages are assigned according to the expression of marker genes BCL6, BLIMP1, MYC, and BCL2 (last columns: Marker). Attractors are given an ID (first column) and a stage name (second column). Color coding of the marker expression (beige: no markers active, orange: MYC and BCL2 active, blue: BCL6 active, green: BLIMP1 and BCL2 active) is used for further analysis as shown in (**b**,**c**). Non-physiological stages are marked as “n.phys.” and blank boxes, see IDs 2, 3, 4. (**b**) Analysis of individual model perturbations on the attractors. For comparison, the WT scenario from (a) is summarized in the first row. Perturbations of the model, specifically ERK ectopic expression (ect) and MYC ectopic expression, are given in two lines below. For MYC, ect-modified attractors occur in DZ, LZ1, and PC that present different marker combinations than the WT, indicated by new colors (legend can be found in Figure 5). (**c**) Detailed view on perturbed attractors, showing the aberrantly active components in red.

In Figure 3a, the eight attractors of the model are listed, all of which are fixpoints and uniquely defined by the combination of inputs. The attractors are assigned to a stage of the GC according to the activity of the marker genes BLIMP1, MYC, BCL6, and BCL2. In the GC reaction, the initiation stage corresponds to the fifth attractor (ID 5), called pre-GC, which describes the process of B-cell homing to the germinal center after activation of the BCR by an antigen. In these cells, survival signaling is active through the PI3K and NF-κB pathway and also MYC is strongly upregulated just before entering the GC in order to provide metabolites for proliferation in the DZ [9]. In the DZ, BCL6 reaches a high level due to IRF4 activity, in order to enable somatic hypermutations by the enzyme AID. In our model, this attractor (ID 6) can only be reached if the BCR is active and additionally there is a signal from IL21 or IL4 to activate STAT. Since T helper cells are mostly absent in the DZ, the cytokines are assumed to be provided by the T cell zone, through which the B-cells migrate to reach the DZ [53].

The light zone presents a very heterogeneous stage in the GC reaction. Depending on the signals that a B-cell receives, it can have different fates, either die, called LZ1 (ID 1); return to the dark zone, called LZ2 (ID 7); or differentiate, leading to the PC attractor (ID 8). In case there are no input signals present, the B-cell should not express any survival or differentiation markers (ID 1). For the dark zone return, an upregulation of MYC is required to allow for proliferation, where MYC activation results from BCR and CD40 input [32]. Only a fraction of cells in the germinal center differentiate into plasma cells (ID 8), where high IRF4 levels are required in combination with STAT3 to induce the transcription of the B-cell marker BLIMP1. BLIMP1 is a transcription factor itself that promotes plasma cell genes, therefore, its upregulation marks a point of no return [54]. In the model, cells that receive BCR, CD40, and additionally IL21/4 signals are able to express BLIMP1 and become plasma cells.

There are three input combinations that lead to attractors in the model that we consider non-physiological (ID 2, 3, 4). Input combinations with active CD40 and/or IL21/4 but inactive BCR are considered non-physiological, since binding of an antigen by the BCR is necessary for the B-cell to process and present the antigen to a T cell. This presentation in turn activates the T cell and finally signals back to the B-cell via CD40 or IL21/4 (or both). As a consequence, those attractor combinations are not considered for the analysis.

### 3.3. Simulating the Effect of Genetic Lesions and Drug Treatment

Having established the model that describes the different GC stages as attractors, we next used the model to simulate the effect of perturbations and predict the impact of inhibitors. For this aim, a copy-number loss or ectopic expression of a gene was implemented into the model by decreasing or increasing the activity level of an affected component [55] (Section 2.2). For example, we can simulate an ectopic expression of ERK by substituting the function from ERK=RAF ∧ ¬ STAT to ERK=1. Consequently, ERK is always active and independent from the inputs and the edges from RAF and STAT. This new model has different attractors than the unperturbed model, which indicates abnormal behavior of a cell carrying such a perturbation. Figure 3b visualizes this change in attractors for the GC stages, where the activity pattern of the marker genes is color coded. In the example of an ectopic expression of ERK, the only attractor that changes is the DZ attractor (see Figure 3b, second line), which now shows MYC and BCL2 activity instead of BCL6 (Figure 3c). However, if MYC is perturbed, which is often the case in DLBCL, the behavior of the model differs in three out of five attractors, shown in the second line of Figure 3b and in detail in Figure 3c. Again, the DZ attractor changes, but to a qualitatively different attractor than present in the wild type, since MYC and BCL6 are both active. This combination can be interpreted as a possible oncogenic state, since BCL6 no longer inhibits MYC to limit the proliferation while undergoing somatic hyper mutations. The simulation shows that a cell carrying a genetic lesion has the risk to transform into a cancer cell, in this case, in a specific environment with active BCR and IL21/4.

**Figure 4:**
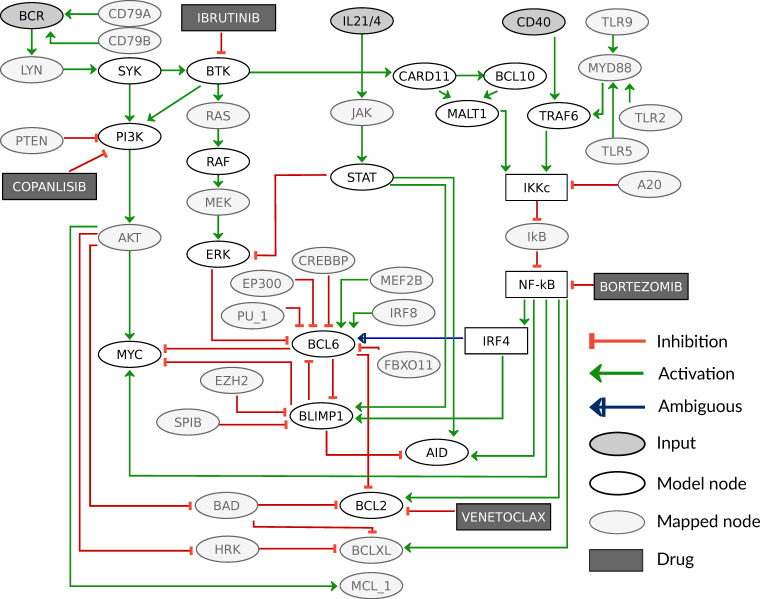
Extended graph of the model including additional components with mapped genetic lesions and simulated drugs. Additional nodes are indicated by light grey color and inhibitors are shown in dark grey boxes. The components CARD11, BCL10, and MALT1 that form the CBM complex are shown separately here, but are mapped on the component CBM in the model. Please note that an activation in the model in Figure 2 might be based on two consecutive inhibitions in the extended model version, e.g., IKKc activation of NF-κB.

Our model describes signaling and gene regulatory processes that are important for B-cell development and therefore already contain a number of components that are known oncogenes or tumor suppressors in DLBCL, such as BCL6 or BCL2. In order to cover more patients, we expand the coverage for genetic lesions that can be implemented in the model. For this aim, the involved pathways are extended and regulators of central genes are added that are present as genetic lesions in patients of the data set [7]. We found 54 genes that were either part of a modeled pathway or a direct regulator of a marker gene, as shown in Figure 4. In the figure, a few components are lumped, e.g., STAT maps STAT1, STAT3, STAT5, and STAT6, while IκB and NF-κB cover all members of the respective family. Perturbations in nodes that are not in the model are mapped to the corresponding model component (details Section 2.2). For example, an amplification in the oncogene HRAS was mapped as a constant activation to the downstream kinase RAF. A LOF mutation in the gene TNFAIP3, encoding for A20, is implemented as GOF in IKKc, since A20 is a tumor suppressor connected by a negative edge to IKKc. This perturbation was shown experimentally to cause a constitutively active NF-κB pathway [56]. Other examples are IRF8 and MEF2B, which are known to activate BCL6 and have been reported as oncogenes in DLBCL; however, their upstream regulation is not well described [3]. Therefore, we included them by mapping their perturbations directly to BCL6. Moreover, functionally similar components were mapped on one representative node. This was done for the BCL2 family, where pro-survival factors BCL2, BCLXL, and MCL1 have a comparable molecular effect and are therefore represented by BCL2. Lastly, we implemented the effect of four different drugs by decreasing the activity of their targets, where Ibrutinib inhibits BTK, Copanlisib inhibits PI3K, Bortezomib inhibits NF-κB, and Venetoclax inhibits BCL2. All these perturbations can be simulated separately or in every combination. Each combination creates a new model that can be simulated and analyzed.

### 3.4. Patient-Specific Models Often Present a Shift in Attractors

After mapping individual genetic lesions as perturbations to the model, we can include the specific combination of perturbations for each patient. From the 304 patients in the data set of Chapuy et al. (2018) [7], 164 patients carried at least one genetic lesion in a gene that was covered in our model. Overall, we found 1–10 perturbations per patient. The Oncoprint in Figure A1 shows that these patients have BCL2 as the most frequent lesion, which is reported to be the most common genetic lesion in DLBCL. BCL2 is altered very distinctly in ABCs and GCBs, with ABCs having gene amplification and GCBs mutations [57], which is clearly reflected in Figure A1. In the frequency of mapped perturbations per patient, there is no prevalence in GCBs or ABCs.

For the analysis, a patient-specific set of genetic lesions was implemented in the model using the CoLoMoTo notebook [27], resulting in a specific model for each patient. The attractors for each GC stage are given and color-coded according to the combinations of expressed marker genes. This allows not only to identify qualitatively abnormal attractors, which is marker gene combinations that are not present in the wild type, but also to observe markers in the wrong stage of the germinal center.

In Figure 5, the attractors for a selection of patients (58 out of 164 for better visibility) are shown. The respective components carrying an alteration are given on the left hand side. In the first row, the WT behavior from Figure 3 is depicted for comparison. Some patient-specific models show a very similar behavior to the WT, while other models differ in one up to every attractor. We here discuss a few examples in more detail: For the patient model 131, only a CD79B alteration was found resulting in a constitutively active BCR (see Figure 4). The attractors Pre-GC, DZ, LZ2, and PC all have active BCR as input (Figure 3a), thus they appear unperturbed compared to the WT (Figure 5). However, the LZ1 attractor requires an inactive BCR, which does not exist anymore for this model, because of the CD79B alteration. As a result, the LZ1 attractor is lost. Patient model 73 has an alteration in MCL1, which is mapped as a GOF to BCL2, resulting in two changed attractors. The LZ1 attractor is changed from no active markers to active BCL2, resulting in a potential escape of the selection process. Moreover, the DZ attractor normally has active BCL6, which inhibits BCL2, but due to the genetic lesion in BCL2 this inhibition is lost, leading to BCL6 and BCL2 being active together.

Generally, the more alterations per patient are mapped, the stronger is the difference in the model behavior, with exceptions. Patient 29 has alterations in BCL2 and STAT as well as EZH2, which is mapped to BLIMP1. Here, all but one of the attractors are different to the wild type, showing BCL6 and BCL2 active together in DZ and Pre-GC, a loss of the attractor in LZ1 and loss of the PC stage. Models 47 and 34 also show differences to the WT, where model 47 has a constantly strong (level 2) activity of NF-κB caused by a perturbation in TLR2. Together with a PTEN LOF resulting in constitutively active PI3K, MYC is always active unless IL21/4 drives the cell towards BLIMP1 expression (Figure 5). In model 34, there is a medium level of constitutive NF-κB (level 1) due to a perturbation in CARD11 as well as an amplification in MYC and BCL2 leading to aberrant marker combinations in the DZ and LZ1 with BCL6, MYC, and BCL2 activity. Moreover, the PC stage has a non-physiological combination of BLIMP1 with BCL2 and MYC active (Figure 5).

All the aforementioned patient-specific models still show a sensitivity to the input, meaning that the marker gene expression depends on the stage and therefore the environment of the cell. For other models, e.g., 27, 60, and 85, this is lost completely, implying a similar marker gene activity in every GC stage. In these examples, the key feature is a constitutive BCL6 activity, e.g., for model 60, this is caused by a mutation in MEF2B. Although this patient also has an additional mutation in TLR2, leading to a high NF-κB level, this has no effect on the marker genes in the model. This is due to the central role and dominant effect of BCL6, which inhibits all of the other three marker genes. As soon as BCL6 is active, it inhibits MYC, BLIMP1, and BCL2, unless there are perturbations directly in these genes causing a disruption of the inhibition. This can be observed in model 85, which has a constitutive BCL2 activation in addition to a BCL6 perturbation via EP300 (see Figure 5). Finally, model 27 has what is described as a very aggressive combination of genetic lesions with constitutively active BCL6, BCL2, and MYC. In this patient, BCL2 and MYC are directly altered, EZH2 activation inhibits BLIMP1, and MEF2B as well as CREBBP cause active BCL6. While the perturbation in PTEN mutation leads to permanently active PI3K, the marker gene activity is unchanged in our model, since all PI3K targets are perturbed and therefore disconnected from upstream signals.

Finally, model 92 is one of a few models with a LOF in NF-κB due to an amplification in IκBε (Figure 5). The model still responds to inputs via PI3K signaling, leading to BCL2 activity, but MYC and BCL6 cannot be active anymore. Since the next downstream component of NF-κB signaling, IRF4, is also perturbed as GOF, BLIMP1 can still be activated. Here none of the expected oncogenes are active, but the model clearly shows abnormal attractors.

### 3.5. Oncogenic Attractors Are Quite Heterogenous

The patient-specific models showed a variety of new attractors with variable marker gene expression. We then analyzed which of these attractors have to be considered as oncogenic. Based on literature information, oncogenic states are defined as abnormal marker combinations, e.g., expression of BCL6 in combination with BCL2 and/or MYC is an aggressive cancer state. Another possibility is a marker gene expression at the wrong stage, e.g., expression of BCL6 outside of DZ or MYC expression outside of Pre-GC and LZ2. In detail, the activity of BCL6 as a DNA damage response inhibitor should be limited to the DZ and is especially problematic when combined with MYC as a proliferation driver and BCL2 as an anti-apoptotic regulator. Both of these components are normally inhibited by BCL6 and are well described to escape this inhibition in DLBCL through structural variants, with even poorer prognosis in double or triple hit patients [58,59]. In Figure 5, darker colors are used to visualize multiple marker gene activities. Finally, a loss of specific attractors can mark an oncogenic state; important examples are the loss of the PC attractor and the loss of the LZ1 attractor. The PC attractor represents the exit of the GC, whose avoidance is described in the literature as one feature of DLBCL, either by genetic lesions of BLIMP1 or its regulators [1]. The loss of the LZ1 attractor as the potential apoptotic state in the model is of special interest, too. The LZ1 attractor represents the state, where the cell does not receive any input from the receptors and therefore does not express any marker genes. The LZ1 attractor is lost for the majority of models, that is for 154 out of 164 patients. Overall, 157 patient-specific models show an oncogenic behavior in their attractors according to the discussed criteria.

**Figure 6:**
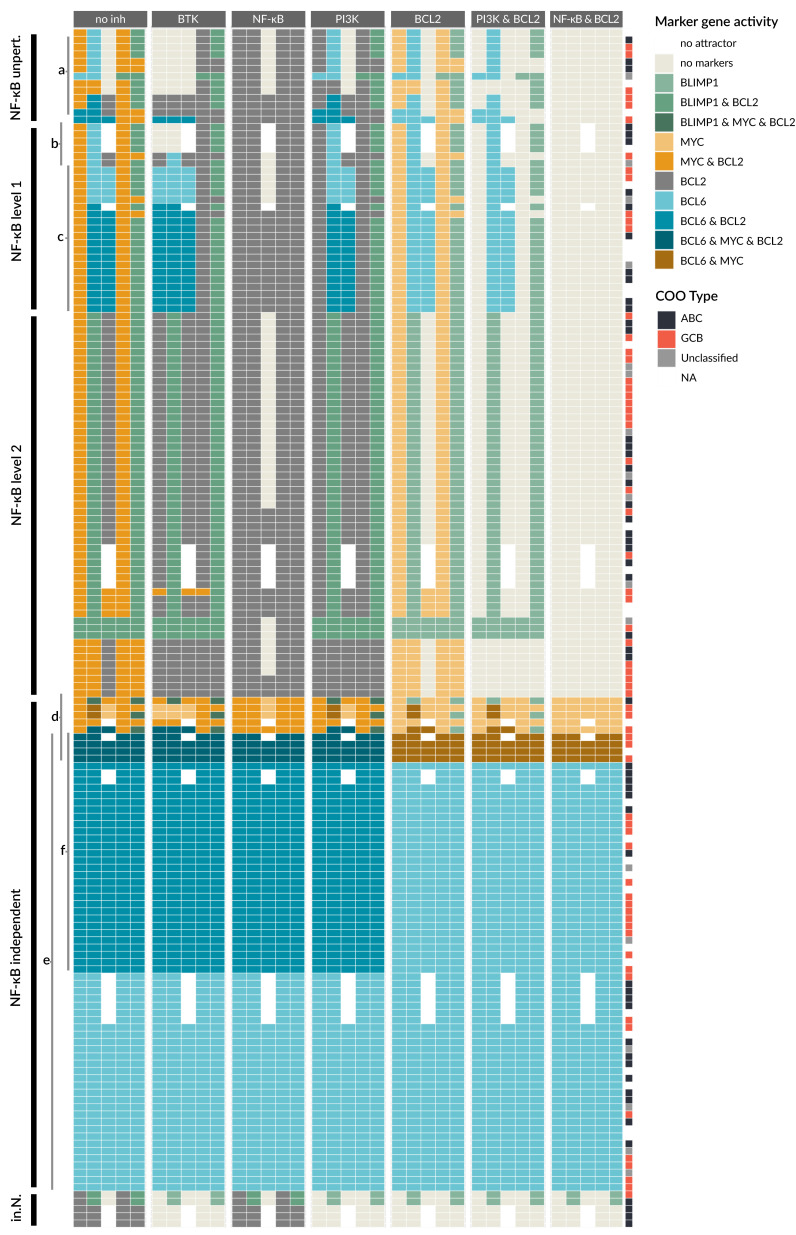
Patient-specific models show differences in sensitivity towards drugs in attractor behavior. Attractors of all 164 patient-specific models are grouped in five classes: NF-κB unperturbed (‘NF-κB unpert.’), NF-κB level 1, NF-κB level 2, NF-κB independent, and inactive NF-κB (‘in.N.’). The first block shows attractors in the different GC stages without a drug (‘no inh’), while the other blocks have an additional perturbation simulating the effect of different individual and combined inhibitors, e.g., BTK inhibitor (block 2) or PI3K and BCL2 inhibitors (block 6). Letters a–f indicate groups discussed in the text. Additional information is provided in Figure A2.

The cancer type DLBCL, especially ABCs, is often described to be driven by or addicted to NF-κB signaling [60], and many mutations occur in the regulation of this pathway. We next wanted to stratify patients based on the influence of NF-κB signaling on the markers. In detail, we determined the dependency of the attractors on NF-κB activity for all patient models. We were able to identify five different classes, where four are NF-κB dependent: NF-κB unperturbed, NF-κB level 1, NF-κB level 2, and inactive NF-κB, and the last class is NF-κB independent (Figure 6).

Most patient-specific models have NF-κB-dependent marker gene expression, where we distinguish between models without perturbations in the NF-κB pathway and those having gain- or loss-of-functions. The NF-κB unperturbed models have no genetic lesions in NF-κB and its regulators. Generally, we observe in this group a very low number of implemented genetic lesions, which are in the MAPK, PI3K, or STAT pathways. The number of changed attractors in these models is low (Figure 6a, first block).

A large group of patient-specific models has a perturbation that causes a constitutive activation of NF-κB. This activation can be due to genetic lesions in the receptors such as TLR2, but also CBM complex component mutations or amplifications directly in NF-κB family members, such as in cREL. As a result, all of these patient models show a loss of the LZ1 attractor. Since our model distinguishes between two different activity modes of NF-κB signaling, reaching either a level 1 by BCR signaling or a level 2 by CD40 activation, alterations can cause level 1 or 2 activity of NF-κB as well. In Figure 6 (first block), we can observe that the marker gene expression of the patient-specific models is mostly similar within the NF-κB level 1 and level 2 group, but is more distinct between these groups. The NF-κB level 1 group shows an increase in attractors with BCL6 activity in LZ1 (Figure 6). For level 2 models, BCL6 generally cannot be active anymore, since it requires an IRF4 activity of level 1 and therefore an NF-κB activity of level 1 (see Table 1). Instead there is a PC-like attractor with active BLIMP1 in the DZ stage or active MYC in the DZ and PC.

There is a group of patient-specific models with perturbations in the targets of NF-κB causing an NF-κB independency (Figure 6, ‘NF-κB independent’). Here, the state of NF-κB does not affect the marker gene activity. This group makes up for approximately a third of the 164 patients and interestingly shows a similar prevalence for ABC and GCB DLBCL. The largest sub group of patients in this class harbors a BCL6 perturbation (Figure 6e). A smaller group of patient models is MYC driven (Figure 6d), with four patient models showing both MYC and BCL6 perturbations.

Finally, the group ‘inactive NF-κB’ (in. N.) encompasses a small number of patients with inactivating perturbations in NF-κB, for example by amplification of the NF-κB inhibitor IκBε. These models express very few marker genes in their attractors (see Discussion).

### 3.6. Drug Simulation Shows Resistance for BCL6 Perturbed Samples

Finally, we used the patient-specific models to investigate the effect of inhibitors, with a focus on inhibitors that interfere with the signaling pathways covered by our modeling approach. Therefore, we perturbed each patient-specific model by single inhibitors or selected inhibitor combinations. The results are shown in Figure 6 (second to seventh block), demonstrating that each inhibitor strategy yields a new set of attractors per patient. The aim of drug treatment is to drive a cancer cell into apoptosis. Since this cell fate is not explicitly represented in our model, the state ‘no marker’, where BCL2 and all other marker genes are inactive, is taken as a surrogate for a state where pro-survival signaling is lowered and therefore apoptosis is more likely. The challenge is to identify an inhibitor that shifts as many attractors as possible to the ‘no marker’ state.

In Figure 6, we can observe that the NF-κB-independent class either does not respond to any inhibitor at all, such as BCL6-driven patient models, or the inhibitor does not have enough impact to reach the ‘no marker’ state (Figure 6e). Here, impact means the capacity to turn off as many marker genes as possible. For the BCL6-driven samples, the inhibitors for BTK, NF-κB, and PI3K all act upstream of BCL6 and therefore have no effect on the marker gene activity. Only the BCL2 inhibitor blocks BCL2 in models that have a GOF in this gene (Figure 6f), but since BCL6 stays active it is still categorized as oncogenic. For patient models with activated MYC (Figure 6d), inhibition of BTK, NF-κB, and BCL2 can show a small effect (blocks 2, 3, 5), and the combination of NF-κB and BCL2 inhibitors in Figure 6 (block 7) has the strongest impact. However, in these models, MYC always stays active independent of inputs and inhibitors.

The patient models in the other groups (Figure 6; NF-κB unpert., NF-κB level 1, NF-κB level 2) are to some extent sensitive to the inhibitors. The inhibitor BTK (block 2) is generally able to block NF-κB, MAPK, and PI3K pathway activation after BCR activation and can therefore efficiently block survival signaling. This effect can be observed in the NF-κB unperturbed group (Figure 6a, block 2) and models in the NF-κB level 1 group with perturbations in the BCR (Figure 6b, block 2). The models of these patients show a ‘no marker’ attractor for Pre-GC, DZ, and LZ1, but not for LZ2 and PC where CD40 is active. The BTK inhibitor has a larger impact than NF-κB, PI3K, or BCL2 inhibitors that show fewer ‘no marker’ attractors for these models. However, this benefit changes for NF-κB level 1 models with perturbations in the CBM complex (Figure 6c, block 2). Here, BCL6 is active in the DZ and LZ1, where inhibiting BTK leads to an additional activation of BCL6 in the Pre-GC setting. This is due to the fact that ERK is normally repressing BCL6 upon BCR activation. In this case, BTK cannot block NF-κB signaling since the perturbation is downstream of BTK, but it still blocks ERK, allowing for BCL6 activity. For these patient models, an NF-κB inhibitor is a better choice, since it can block all marker gene activity except BCL2 (Figure 6c block 3), in which case a combination of NF-κB and BCL2 inhibitors is the most effective (Figure 6c block 7).

For models with NF-κB level 2 perturbations, BTK, NF-κB, and PI3K inhibitors all stop MYC activity, with one exception. One patient has a PTEN and TLR2 perturbation that causes BTK inhibitors to have no effect, since both PI3K and NF-κB signaling are driven by perturbations independent of BTK. Moreover, all patient models that show BLIMP1 activity benefit from NF-κB inhibitors (Figure 6, block 3), while BTK and PI3K inhibitors do not affect BLIMP1 activity. The opposite is true for the few patients with no NF-κB activity (Figure 6 in.N.), where only BTK and PI3K inhibitors show a beneficial effect.

The single BCL2 inhibition also has a limited impact, see Figure 6 (block 5). However, for BCL2 inhibition in combination with BTK, PI3K, or NF-κB inhibitors, the attractors of many patient models can be shifted to the ‘no marker’ state. In particular, the combination of NF-κB and BCL2 inhibitors can shift all attractors of all patient models in the NF-κB unperturbed, NF-κB level 1 and level 2 to the ‘no marker’ attractors (Figure 6 block 7), suggesting that this combination can be beneficial for a large group of patients.

## 4. Discussion

In this paper, we develop the first patient-specific models for DLBCL by combining a mechanistic description of critical processes in B-cell development in the GC with published data of genetic lesions from individual patients. The approach demonstrates a heterogeneity of attractors in the individual patient-specific models. For the description of the behavior of a model, we specifically did not summarize the attractors of the patient-specific models but investigated the effect of perturbations on the full physiological attractor list. This provides critical detail for the simulation of inhibitor effects, where we observed model-specific sensitivity even within patient model groups with similar pathway perturbations, e.g., NF-κB level 1 models.

In general, by applying our modeling framework, we were able to develop models for more than half of the patients from the Chapuy et al. study [7] (164 out of 304), indicating that we capture many of the relevant processes with our underlying model. However, there are also patients that have no perturbations in the 54 genes covered by the model and several patient models showed a non-oncogenic behavior by either presenting the wild type attractor pattern or attractors that did not have any of the known marker genes active. For all these cases, we expect that the genetic lesions driving DLBCL are not captured in our current model. An important example is the p53 pathway, with p53 as a common driver of DLBCL that is not yet included into the model but could be subject to future extensions based on existing models [61,62]. Given the importance of the NF-κB pathway, more details of the pathway can be included, e.g., regulatory feedbacks, processes of the non-canonical pathway, and distinct roles of NF-κB subunits [50,63,64,65]. In addition, it would be advantageous to expand the description of survival and apoptosis processes to obtain a more direct read-out for cancer development. Here, crosstalk between pathways will be of specific interest, e.g., the STAT pathway is also involved in apoptosis regulation in the GC by upregulating the expression of the pro-apoptotic factor BIM [66].

Moreover, additional investigations of receptor stimulation experiments would allow to add more detail to the model. Specifically, testing which receptor activation is sufficient and necessary to trigger the dark zone expression program or to decide if a B-cell develops into a plasma cell instead of returning to the dark zone. For example, whether CD40 is critically necessary for PC differentiation is controversial. There have been in vitro experiments showing that strong BCR and IL21 stimuli can cause PC differentiation [48]. However, some studies suggest that this is not physiological in the LZ since the cells mostly have an internalized BCR. In our model, we inferred the receptor activity from the known marker gene states and their connection via signaling pathways. Thereby, we can show that IL21/4 is required in the DZ to suppress ERK, otherwise BCL6 could not be active. Also, it is not clear if the BCR stimulus alone is sufficient for B-cell entry into dark zone or an additional CD40 stimulus is required. In our model, the combined BCR and CD40 activation leads to the same marker expression as BCR stimulation alone. Therefore, both inputs could be necessary for DZ return as well as initial entry into the GC (Pre-GC) as described by Schwickert et al. [53]. There is a lack of experimental data exploring the behavior of a GC B-cell after stimulation of individual receptors as well as combinations at a specific time point.

We found a larger number of patient models that still show the plasma cell attractor, which a cancer cell never reaches. This is due to the implementation of perturbations for the NF-κB pathway, where perturbed components adopt the highest value possible. For perturbations in the ternary components IKKc, NF-κB, and IRF4, this means that they automatically adopt the value 2. Here, quantitative information for different mutations would be valuable to test whether this is necessarily true, or if these perturbations could cause an NF-κB activity of level 1.

In general, there are different strategies for patient stratification and different types of models can be employed. For example, statistical models are used to classify patients into groups based on genomic, transcriptomic, and/or clinical data to create prognostic scores for DLBCL patients [7,8,67,68]. On the other hand, mechanistic models of pathways allow to simulate the effect of perturbations on a molecular level. For that aim, several computational models for B-cell development have been created, including logical models as well as ordinary differential equation (ODE) models. Sciammas et al. (2011) [47] concentrated on the kinetic regulation of the marker genes and Rodríguez Martínez et al. (2012) [49] investigated the effect of perturbations of specific marker interactions in lymphomagenesis. Roy et al. (2019) [50] focused on B-cell differentiation processes downstream of NF-κB signaling in a population-based model, with a prediction for the role of RELA and cREL in DLBCL. Du et al. (2017) [69] modeled the upstream regulation of NF-κB by BCR signaling in DLBCL using ODEs. The model was fitted to DLBCL cell line data and used to predict drug sensitivity and synergies for this cell line.

In our model, we combined the upstream regulation of NF-κB by BCR and CD40 with the regulation of marker genes for B-cell differentiation. In order to include the signaling connection between BCR and NF-κB via the CBM complex, which is critical to describe the effect of genetic alterations in the BCR signaling, we extended the model of Mendez and Mendoza (2016) [51]. Thereby we are able include patient-specific genetic alterations within the modeled pathways in a more comprehensive way. Recently, several patient-specific modeling approaches have been developed using logical-based ODEs or stochastic logical models [16,70], showing promising results for melanomas, leukemia, or breast cancer [15,17,19]. However, those approaches require additional quantitative data for each patient such as expression or proteomic data to train the models.

Overall, we could demonstrate that using our model approach allows to integrate mechanistic pathway knowledge and genome-scale characterization of patients. It shows that the development of individual patient-specific models delivers a differential picture that can be beneficial, especially for drug predictions.

## Figures and Tables

**Table 1:** Logical functions for components with more than one regulator. The logical operators are ∨ (OR), ∧ (AND), and ¬ (NOT). If the level of the ternary components (IKKc, NF-κB and IRF4) is not specified, it implies their activity for level 1 and 2.

Component	Function	Reference
PI3K	SYK ∧ BTK	[29]
MYC	PI3K ∧ NF-κB ∧ ¬ BLIMP1 ∧ ¬ BCL6	[30,31,32]
ERK	RAF ∧ ¬ STAT	[33,34]
IKKc:1	CBM	[35]
IKKc:2	TRAF6	[36]
BCL6	IRF4:1 ∧ ¬ IRF4:2 ∧ ¬ (ERK ∨ BLIMP1)	[3,34,37]
BLIMP1	IRF4:2 ∧ STAT ∧ ¬ BCL6	[37,38]
AID	NF-κB ∧ STAT ∧ ¬ BLIMP1	[39,40]
BCL2	(PI3K ∨ NF-κB) ∧ ¬ BCL6	[41,42,43]

**Figure A2:**
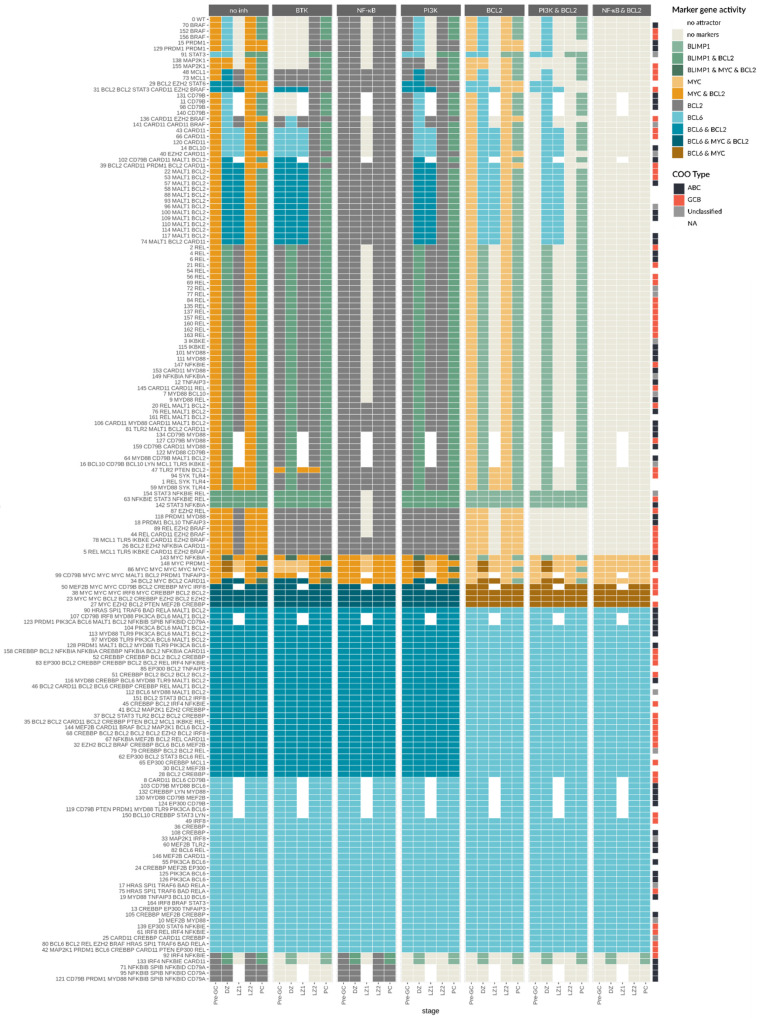
Patient model-specific attractors for individual and combined inhibitors. On the *y* axis the genes that are perturbed for each patient are given, where only those genes that are mapped to our model are shown. On the *x* axis, the five stages of the germinal center reaction from Figure 1 are given for each inhibitor or inhibitor combination described on the top. The color code indicates the activity of markers in each attractor. The column to the right side shows the COO type of the patients according to Chapuy et al. (2018) [7], where ABCs are shown in black, GCBs in red, unclassified in grey, and white shows no information available.

## Data Availability

The model will be uploaded to the GINsim repository.

## Appendix A

**Table A1:** Logical functions for all model components in Figure 2. If the level of a ternary component (IKKc, NF-κB, IRF4) is not specified, it implies activity for level 1 and 2.

Component	Function	Reference
BCR	Input	
IL21/4	Input	
CD40	Input	
SYK	BCR	[29]
BTK	SYK	[29]
RAF	BTK	[44]
CBM	BTK	[44]
TRAF6	CD40	[36]
STAT	IL21/4	[38]
PI3K	SYK ∧ BTK	[29]
MYC	PI3K ∧ NF-κB ∧ ¬ BLIMP1 ∧ ¬ BCL6	[30,31,32]
ERK	RAF ∧ ¬ STAT	[33,34]
IKKc:1	CBM	[35]
IKKc:2	TRAF6	[36]
NF-κB	IKKc	[71]
IRF4	NF-κB	[47]
BCL6	IRF4:1 ∧ ¬ ERK ∧ ¬ BLIMP1 ∧ ¬ IRF4:2	[3,34,37]
BLIMP1	IRF4:2 ∧ STAT ∧ ¬ BCL6	[37,38]
AID	NF-κB ∧ STAT ∧ ¬ BLIMP1	[39,40]
BCL2	(PI3K ∨ NF-κB) ∧ ¬ BCL6	[41,42,43]
